# HLA-G Neo-Expression on Tumors

**DOI:** 10.3389/fimmu.2020.01685

**Published:** 2020-08-14

**Authors:** Maria Loustau, François Anna, Raphaelle Dréan, Martin Lecomte, Pierre Langlade-Demoyen, Julien Caumartin

**Affiliations:** ^1^Invectys, Paris, France; ^2^Molecular Virology and Vaccinology Unit, Virology Department, Institut Pasteur & CNRS URA 3015, Paris, France; ^3^Molecular Retrovirology Unit, Institut Pasteur, CNRS, UMR 3569, Paris, France

**Keywords:** HLA-G, immune checkpoint, hematopoietic tumors, solid tumors, immunotherapy

## Abstract

HLA-G is known to modulate the immune system activity in tissues where physiological immune-tolerance is necessary (i.e., maternal-fetal interface, thymus, and cornea). However, the frequent neo-expression of HLA-G in many cancer types has been previously and extensively described and is correlated with a bad prognosis. Despite being an MHC class I molecule, HLA-G is highly present in tumor context and shows unique characteristics of tissue restriction of a Tumor Associated Antigen (TAA), and potent immunosuppressive activity of an Immune CheckPoint (ICP). Consequently, HLA-G appears to be an excellent molecular target for immunotherapy. Although the relevance of HLA-G in cancer incidence and development has been proven in numerous tumors, its neo-expression pattern is still difficult to determine. Indeed, the estimation of HLA-G's actual expression in tumor tissue is limited, particularly concerning the presence and percentage of the new non-canonical isoforms, for which detection antibodies are scarce or inexistent. Here, we summarize the current knowledge about HLA-G neo-expression and implication in various tumor types, pointing out the need for the development of new tools to analyze in-depth the HLA-G neo-expression patterns, opening the way for the generation of new monoclonal antibodies and cell-based immunotherapies.

## Introduction

**Figure 1:**
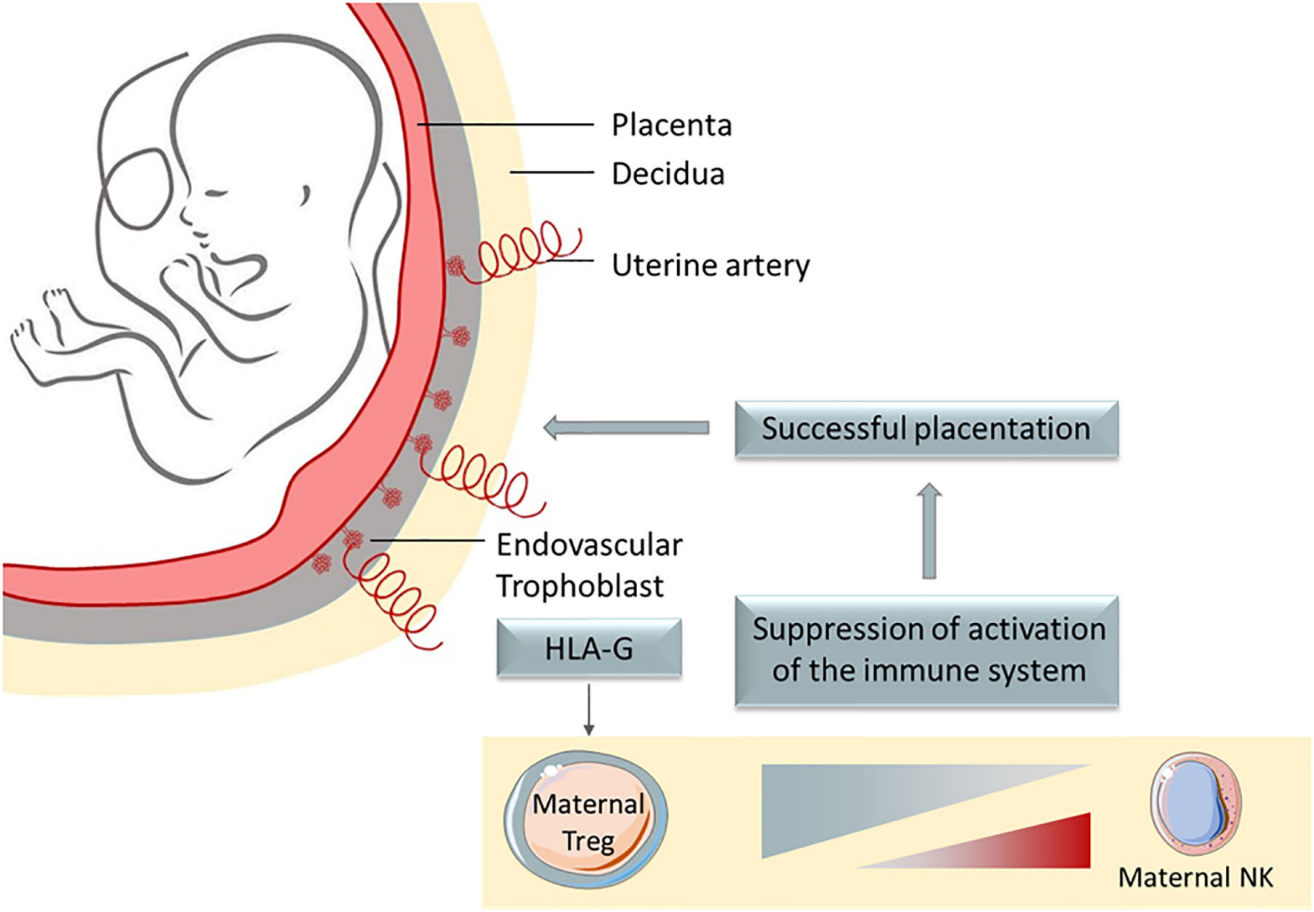
HLA-G is a potent immune escape mechanism, whose main physiological role is to protect the semi-allogenic fetus from mother' immune system, being expressed on extravillous trophoblast that invades the decidua.

Fetus and tumor development are closely related since they are both characterized by a rapid tissue proliferation, associated to a high expression of telomerase (1, 2) and expression of anti-apoptotic factors like survivin (3, 4). Placenta and tumor development is accompanied by angiogenesis induced by proteins of the VEGF family (5, 6) and favored by hypoxia (7). Strikingly, placenta and tumors are protected from the immune system through common immune escape mechanisms. Particularly, the induction of a tolerogenic microenvironment was demonstrated, involving the expression of inhibitory immune checkpoints inducing suppressive macrophages, dendritic cells (DCs) and regulatory T cells (T_regs_). Among the pool of inhibitory checkpoints shared between the placentation process and the tumor development, HLA-G is emerging as a potent immune escape mechanism (Figure 1).

HLA-G is a non-classical MHC class I molecule first determined to be expressed on extravillous trophoblast that invade the decidua (8–11), similarly to the invasive growth process observed for tumors (12). Despite being restrictively expressed on healthy tissues, HLA-G was reported to be neo-expressed in several pathological contexts, especially during tumor development (13, 14). HLA-G neo-expression is always associated with a bad prognosis for patients.

Contrary to the classical MHC, HLA-G is characterized by a low polymorphism and tolerogenic functions. HLA-G can be expressed under, at least, seven isoforms. These are the product of alternative splicing of a single primary transcript of RNA (15): four membrane isoforms (HLA-G1, HLA-G2, HLA-G3, and HLA-G4) and three soluble isoforms (HLA-G5, HLA-G6 and HLA-G7). HLA-G1 and HLA-G5 isoforms present the typical structure of MHC classical class I molecules: one heavy chain composed of three globular domains, associated or not to β-2-microglobulin (β2M). The other isoforms are shorter, with one or two globular domains and none is associated with the β2M (16–18). HLA-G exert its biologic tolerogenic function as a ligand by binding its specific receptors: ILT2 (LILRB1, CD85j), ILT4 (LILRB2, CD85d) et KIR2DL4 (CD158d) (19). HLA-G is the ligand of highest affinity for ILT2 and ILT4 receptors. Concerning the KIR2DL4 receptor, it is mostly expressed in NK cells, but its interaction with HLA-G and its inhibitory function remain controversial (20, 21). ILT2 and ILT4 belong to the leukocyte immunoglobulin (Ig)-like receptor family (LILRs), particularly to the inhibitory group: LILRBs, composed of 2 to 4 extra-cellular globular domains and 2 to 4 cytoplasmic inhibitory domains “ITIM” (Immunoreceptor tyrosine-based inhibitory motifs). ILT2 is expressed in all immune cell subsets (22), whereas ILT4 expression is limited to antigen presenting cells (APCs) like monocytes, neutrophils, DCs or macrophages. PIR-B is the ortholog of LILRBs in mice, expressed in B cells, DCs, granulocytes and macrophages, exerting the same inhibitory functions (22). ILT2 binds the HLA-G α3 domain, associated with β2M simultaneously (23) while ILT4 binds the α3 domain independently of β2M. Also, HLA-G can form dimers, which increase the avidity of the receptors ILT2 and ILT4 for this molecule (23).

**Figure 2:**
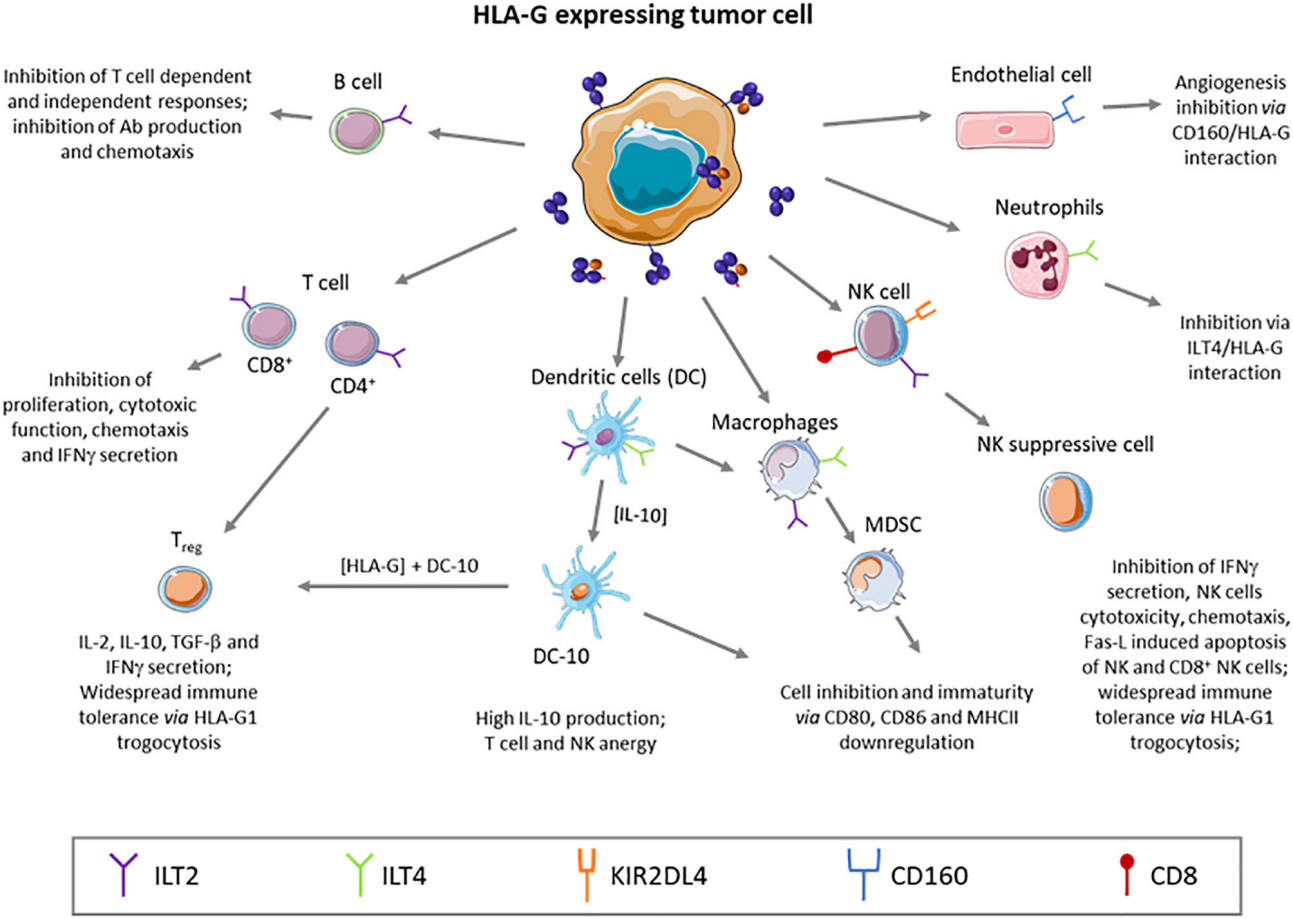
HLA-G is a tolerogenic molecule that broadly regulates the immune system, inhibiting effector cells, or generating regulatory subtypes.

In physiological conditions HLA-G expression has been described in the cytotrophoblast where it plays a major role by protecting the semi-allogenic tissues of the fetus from the maternal immune system. Otherwise, HLA-G is constitutively expressed in immune-privileged tissues like thymic epithelial cells (24, 25), cornea (26), pancreatic islets (27), mesenchymal stem cells (28, 29), erythroblasts or endothelial precursors (30, 31), and some peripheral tolerogenic T and dendritic cells (DC) subsets (32–34). Soluble isoforms have been detected in thymus (24), human first trimester and term placentas *in situ* and *in vitro* (35), plasma (36, 37), cerebrospinal fluid (CSF) (38, 39), in the male reproductive system, in seminal plasma (40), and in the cell culture supernatant of embryos (41–43). However, HLA-G expression can be induced or up-regulated in pathological contexts like (i) cancer (44–46), (ii) auto-immune and inflammatory diseases (47–49), (iii) viral infections (50–52), and (iv) allo-transplantations (53, 54). Indeed, many publications showed the high frequency of HLA-G expression in tumor cells, correlated with clinical background associated with tumor immune escape and bad prognosis (45, 55). HLA-G expression, then, seems to be key for tumors to evade the immune system, even at low rates of expression. Although most of HLA-G immunosuppression function and role in tumor escape studies were performed *in vitro*, HLA-G involvement in tumor escape mechanism was studied and demonstrated *in vivo* in immunocompetent mice through the induction of MDSC (56). Furthermore, Lin et al. evidenced *in vivo* that HLA-G expression was associated with tumor metastasis and with poor survival (57). Inhibition of immune response by soluble HLA-G was also demonstrated *in vivo* (58). Noteworthy, HLA-G expression is induced by hypoxia, typical of solid tumor microenvironment (59). Because HLA-G is found on tumor cells and is rarely observed in healthy tissue, it appears to be an excellent tumor associated-antigen (TAA) to target in immune therapy. Furthermore, HLA-G has been recently defined as a major immune checkpoint (ICP). This molecule is capable of inhibiting not only cytolytic uterine NK cells in the context of pregnancy, but also: (i) cytolytic functions of peripheral NK (60, 61), (ii) cytolytic functions of antigen-specific cytotoxic T lymphocytes (CTL) (62), (iii) alloproliferative response of T CD4^+^ cells (63), (iv) peripheric NK and T cell proliferation (64, 65), (v) B cell maturation and antibody production (66), phagocytic function of neutrophils (67), chemotaxis of NK, T and B cells (66, 68, 69), and (vi) maturation and function of DCs (70). Also, HLA-G was shown to induce the generation of suppressive immune cell subsets (64, 71, 72) (see Figure 2).

Although HLA-G is an MHC class I, it presents rare characteristics combining features of both TAA and ICP, playing a major role in the fine tuning of the immune system equilibrium into a tolerogenic or suppressive microenvironment. HLA-G turns to be a major advantage for tumor cell survival and development. In fact, HLA-G expression has been reported in numerous types of cancer, always associated with more advanced stage and aggressive development of the tumor.

## HLA-G NEO-Expression In Hematopoietic Tumors

The role of HLA-G in hematopoietic malignancies is complex and remains unclear since HLA-G and its inhibitory receptors could be expressed on hematopoietic tumor cells and could inhibit proliferation in such tumors (45). Non-Hodgkin lymphomas (NHL) is a large group of cancers of lymphocytes. There are many different types of NHL which can be divided into aggressive (fast-growing) and indolent (slow-growing) types, composed by either B-cells or T-cells. In NHL, classical MHC molecules and HLA-G expression patterns were shown to be completely altered and correlated to a tumor relapse or transformation (73). It has been postulated that this phenomenon was associated to a deep genetic disorder and rearrangement, inducing HLA-G neo-expression in tumor cells. Chronic lymphocytic leukemia (B-CLL) is a mature lymphoid neoplasm currently categorized as an indolent type of malignant lymphoma. Nuckel et al. reported HLA-G expression on 1–54% of leukemic cells in B-CLL. They determined that patients with 23% or fewer HLA-G-positive cells had a significantly longer progression-free survival (PFS) time than patients with more than 23% of positive cells. Indeed, patients with a weak HLA-G expression showed a higher survival rate (120 months) than those with high HLA-G expression with a survival average of 23 months. Furthermore, humoral and cellular immunosuppression were significantly more prominent in the HLA-G-positive patients' group in comparison to the HLA-G-negative group. Indeed, the survival rate decrease was associated to an immune response deficiency, a CD4/CD8 T cells ratio and immunoglobulins (IgG) reduction and to an increase of secreted soluble HLA-G proteins (74–76).

B-CLL can progress slowly over years, but it eventually transforms into a more aggressive lymphoma such as the diffuse large B-cell (DLBCL) type. The diffuse large B-cell lymphoma (DLBCL) is a B cells cancer and is the most common type of NHL. DLBCL is characterized by its aggressiveness, which can be developed in the lymph nodes or in extranodal sites. DLBCL is the most frequent lymphoma and the most severe. In this type of lymphoma, the expression of HLA-G was determined to be relatively weak. However, the survival rate was directly correlated to the HLA-G expression, increasing from 47.5%, when HLA-G is expressed, to 73.3% in absence of HLA-G expression (77). Expression of HLA-G in classical Hodgkin lymphoma was also independently determined by the groups of Diepstra and Caocci. They both determined a relatively high expression of HLA-G (>54% of expressing tumor cells) in the Reed-Stenberg cells, with particular higher expression in nodular sclerosis (78, 79). However, their results on the HLA-G expression levels were different in the tumor microenvironment (TME).

Cutaneous lymphomas represent the second most frequent extranodal lymphomas and are cancers of lymphocytes primarily involving the skin. Cutaneous lymphomas are classified based on whether they are cancers of B or T lymphocytes, and, respectively, designated as cutaneous T cell lymphoma (CTCL) and cutaneous B cell lymphoma (CBCL). Although being mostly a benign disease, skin clonal lymphocytes can migrate to the nodes resulting in a more severe disease. These cells can persist mostly because of HLA-G and IL-10 secretion (80). All the T or B skin cells were determined to be HLA-G1 mRNA positives, but protein expression level was weaker. A strong correlation between IL-10 and HLA-G expressions was evidenced with a co-expression of these molecules in 73% of the cutaneous lymphoma investigated (81). Furthermore, for T cells, HLA-G protein expression was directly correlated with the tumor grade and stage.

## HLA-G NEO-Expression in Solid Tumors

Tumor development is dependent on its capability to escape from the immune response. According to Dr. Schreiber's 3E theory, three stages define the immune response and the interaction between tumor cells and their microenvironment: elimination, equilibrium and escape (82). The first phase of elimination is characterized by the production of new molecules, derived from oncogenic modifications of the brand-new tumor, and expression on their surface, known as neo-antigens, that are able to induce an efficient response by the immune system. In accordance with the classic immune surveillance theory, those new tumor cells that aren't destroyed in the initial stage, will proliferate, create a primitive tumor and will set up an equilibrium with the immune cells. This equilibrium phase can last months or years, until the tumor becomes able to engage the *escape* phase, where the plasticity of its genome allows it to evolve, change the environment, evade the immune control and spread. At the same time, the immune system might become tolerant or exhausted.

Actually, HLA-G can be involved in these three phases. During the elimination phase, HLA-G can inhibit T and B cells activation, proliferation, cytotoxic function of T and NK cells and can block the DCs and neutrophils functions (60, 62, 68, 76, 83, 84). Throughout the equilibrium phase, HLA-G can downregulate the MHC class II expression on DCs and induce suppressive myeloid cells, favoring the regulatory cell subsets (85). Finally, the escape phase is characterized by a high cell proliferation and, afterwards, a hypoxic environment (86). Hypoxia induces upregulation of V-EGF and HIF-1, and with the latter, HLA-G expression. Also, it was determined that immunosuppressive cytokines, such as IL-10 and TGF-β, are secreted and could favor HLA-G expression and maintenance by positive feedback (87).

HLA-G expression in multiple types of primary tumors has been demonstrated (88). HLA-G can be detected either on the cell-surface of tumor cells or on tumor infiltrating cells (TILs) particularly on lymphocytes, monocytes, macrophages and dendritic cells (DCs) (89–94). HLA-G was demonstrated to be crucial for the tumor development and its expression was specifically associated to malignant transformation (59). HLA-G expression in surrounding healthy tissue has never been detected but its expression in solid tumors has been described, particularly in advanced clinic stages (95, 96). Soluble HLA-G isoforms (sHLA-G) have been detected in patient's plasma with advanced stages and reserved prognostic (91, 95–99). Therefore, the role and functions of HLA-G in tumor immune escape and tumor development is beyond a hypothetical mechanism, its involvement and relevance has been widely documented. This tolerogenic molecule has been described in a plethora of solid tumors.

What brings another level of complexity in the detection of HLA-G and the understanding of its role in cancer progression is the existence of micro-vesicles bearing HLA-G, firstly described in the supernatant of HLA-G positive melanoma cells *in vitro* (100). Intercellular communication through extracellular vesicules (EV) released in the extracellular space or in body fluids is a known mechanism involved in healthy tissues as well as malignancies (101). These structures originate from the cell membrane or are exosomes, and can carry surface proteins, cytokines or growth factors (amongst others). Their role in the immune response modulation has been shown by Abusamra et al. by *in vitro* experiments that evidenced the induction of CD8^+^ T-cells apoptosis by exosomes expressing Fas ligand (102). This observation and the known mechanism of action of EVs suggest that EVs harboring HLA-G could play a role in cancer immune escape, by inhibiting immune cells in the tumor microenvironment or at distal sites. The inhibition of monocyte differentiation and maturation into dendritic cells (DCs) by HLA-G1-bearing EVs originating from kidney cancer cells has been reported (103). Several clinical studies carried out with breast and ovarian cancer patients also support this hypothesis. High levels of HLA-G-EVs in breast cancers patients treated with neoadjuvant chemotherapy (NACT) correlates with a bad prognosis, whereas patients with high levels of free soluble HLA-G had better outcome. Moreover, the level of total circulating HLA-G molecules is not a predictable marker of patient's outcome (104). Similar findings were reported in a study with epithelial ovarian cancer (EOC) patients in which high levels of HLA-G EVs was a marker of inferior clinical outcome (105). Deciphering HLA-G EVs from free soluble HLA-G molecules seems thus to be of crucial importance to improve patient's diagnosis and the understanding of EVs mechanism of action, and investigate their relevance as immunotherapy target.

Renal cell carcinoma (RCC) affects 3% of occidental adults, with an increasing incidence in the last years. There are several subcategories of RCC, the principal being clear cell RCC (ccRCC) that represents 80% of RCC, followed by the papillary and chromophobe carcinoma, 10 and 5%, respectively. Frequently, this cancer is at an advanced stage presenting metastasis at the time of diagnosis, with a low rate of 5 years survival (<15%). In ccRCC, HLA-G mRNA and protein expressions have been strongly described (106–108). These expressions in patients seem to be age or sex independent but are highly related to the ccRCC sub-type. Frequently, there is no correlation between mRNA and protein expression which might be explained by a postranscriptional regulation that blocks translation (109). HLA-G loss of expression at the tumor cell-surface during cell culture could be explained by (i) the absence of transcription factors related to the hypoxic microenvironment or (ii) the lack of several cytokines such as IFN-γ, IFN-α, and IL-10 (110), but (iii) could also be related to a HLA-G isoform switch that could not be detected since antibodies detecting all the isoforms of HLA-G are missing, particularly for those that lack the α1 domain (111). Recently, a heterogeneous expression of immune checkpoints including PD-L1, B7H3, ILT2, and HLA-G in RCC was reported (112). This intratumor heterogeneity was found both at tumor cell and infiltrating immune cell levels in primary RCC (113). Interestingly, target cells' HLA-G expression specifically inhibited cytotoxicity of CD8^+^ILT2^+^ T-cells, but not their CD8^+^ILT2^−^ (PBMC) or CD8^+^PD-1^+^ (TIL) counterparts. HLA-G inhibition was counteracted by blocking the HLA-G/ILT2 interaction showing that CD8^+^ILT2^+^ TILs may therefore constitute a subset of fully differentiated cytotoxic T cells within the tumor microenvironment, independent of the PD1^+^ TILs targeted by immune therapies, and specifically inhibited by HLA-G (114).

Colorectal cancer (CRC) is the 3rd most frequent cancer in the world and the 2nd mortality cause related to cancer. Most of them (96%) are present under the adenocarcinoma form associated to a transformation of luminal epithelial cells of the mucosa of the intestine. It affects mostly aged population (>50 years). It was determined that HLA-G expression was detected in 64% of the tumor samples in the primary site of the carcinoma but HLA-G expression was absent in the surrounding tissue, evidencing HLA-G as a malignant transformation marker (92, 96). HLA-G expression was correlated with advanced stages of the “tumor-node-metastasis” (TNM) classification and a diminution of the survival rate (<3 years) for HLA-G positive patients. However, HLA-G expression in CRC remains controversial. Due to the heterogeneity amongst techniques and technic tools, discordant results were obtained for HLA-G expression in CRC. Furthermore, HLA-G neo-expression was shown to depend on the microenvironment of the primary lesions, yet, absence of TME on metastases cannot be linked to HLA-G neo-expression (115). Thus, primary tumors are mostly associated to an active response expressing immunomodulatory molecules like HLA-G, whereas secondary or metastatic tumors favor a “hiding” strategy, avoiding being detected by the immune system. Co-expression of HLA-G and HLA-E has been described in CRC. Downregulation of expression of the classical MHC class I molecules allow tumor cells to escape from cytotoxic T cells (CTLs) despite rendering them sensitive to the NK response. Inhibition of the NK response is related to the upregulation of HLA-G and HLA-E which mediates the inhibition of NK cells through ILT2 and NKG2A receptors, respectively (83, 116).

Esophageal carcinoma is the 8th most frequent cancer in the world and the 4th cause of death related to cancer. The most frequent type is the esophageal squamous-cell carcinoma (ESCC) (90%). HLA-G expression extended from 65 to 90% of ESCC cases and was related to advanced stages of TNM (117–119). The lower survival rates were shown to be related to tumor cell infiltration in the lymph nodes and to the HLA-G expression. The mRNA studies performed indicated that the most frequent isoforms present were HLA-G1 and -G5 in the primary tissue, but were absent from surrounding tissue, and HLA-G5 was detected in patients sera (118).

Gastric cancer is the 5th most frequent cancer and causes more than 700 000 deaths per year in the world, being the 3rd cause of death in cancer. Usually, at the time of diagnosis this cancer is advanced and frequently presenting metastasis. 90–95% of gastric cancer are adenocarcinoma with a gastric superficial mucosa cells origin. High HLA-G expression was reported with 73% of cases, from which 75% presented high expression levels (>50% of cells expressing HLA-G) (97). This expression is exclusive to the primary tumor, without no expression detected on the environmental tissue, and is related to the localization of the tumor, with higher expression in the cardia. Higher HLA-G expression is correlated with advance stage of the disease, the tumor lesion depth, the node invasion and the decrease of the survival rate. Several other groups have demonstrated corresponding results that HLA-G expression in tumor cells correlates with sHLA-G in patients' sera and higher infiltration of T_reg_ CD4^+^ CD25^+^ FoxP3^+^ (120, 121). Also, Du et al. demonstrated that co-culture of PBMCs and SGC-7901 (a gastric cell line), transfected with HLA-G1, can induce an immune regulatory phenotype with an increase of IL-4 and IL-10 secretion and a decrease of IFN-γ secretion (121).

Pancreatic cancer is a relatively rare cancer (<2% of cancers). This adenocarcinoma is very aggressive, associated with a very bad prognosis. Pancreatic cancer is generally of endocrine origin, but it metastases easily, frequently to the liver, stomach and lungs. Zhou et al. have studied HLA-G expression in this cancer, and they have determined that 39.2% are HLA-G positive (122), depending on the tumor grade, increasing from T1-2 to T3 stages. HLA-G expression is correlated with a decrease of infiltrating T cells (TILs) CD3^+^. Other groups confirmed the HLA-G expression in pancreatic adenocarcinoma ranging from 63 to 66% of tumors (123, 124). Xu et al. correlated the HLA-G expression with more aggressive characteristics, a more advanced stage (TNM II), an extra-pancreatic infiltration (T3 stage) and a lymphatic nodes engagement (124). Also, plasmatic sHLA-G was higher in pancreatic cancer patients in comparison to the controls and inversely proportional to CD8^+^ CD28^+^ peripheral T cells.

Hepatocellular carcinoma (HCC) is the most common type of primary liver cancer in adults and the fourth most common cause of cancer-related death worldwide. HCC is usually caused by a chronic disease (infection or cirrhosis). HLA-G expression was determined to be present in 66.7% of the cases and correlates with a more advance TNM stage: 41.9% in stage II to 71.4% in stage III (125). HLA-G expression is associated with an increase of the T_reg_/CD8^+^ ratio and relapse occurs after ablation or resection. Other groups observed similar results (126, 127). Cai et al. indicated that the HLA-G expression remained diffuse and intracellular, detected HLA-G isoforms were essentially the HLA-G1 isoform (detection through WB) without the presence of the HLA-G5 isoform. Yet, sHLA-G was detected in patients' sera (125). This could be explained by the shedding of the HLA-G1 membrane isoform or by the expression of this molecule by other cells like monocytes as previously observed in melanoma and lung cancer. It was hypothesized that the expression of HLA-G could be sustained by the microenvironment of the primary tumor in agreement as observed in other type of cancers (107, 109, 115, 128) and with the 3E theory that points out that the metastatic sites of a cancer should present a totally different microenvironment from that of the primary tumor (82).

Thyroid nodes are cancers of the thyroid affecting 50 to 70% of the adult population which are mostly benign. This neoplasia presents a variable evolution and is constituted by 3 histologic sub-types: papillary thyroid carcinoma (PTC), follicular thyroid cancer (FTC) and anaplastic thyroid cancer (129). It has been demonstrated that HLA-G expression is crucial for the development of these cancers. Indeed, HLA-G expression is absent in non-pathological histologic tissue (130) whereas HLA-G expression is determined to be present in all thyroid tumors. A strong expression of HLA-G (>50% of HLA-G^+^ cells) was observed in 80% of PTC and 79% of FTC but also in benign lesions. However, HLA-G expression was not correlated with cancer relapse, metastasis, node invasion or with mortality rate. It was proposed that HLA-G was necessary for cancer genesis given its pre-tumoral expression. Other groups confirmed this expression (131), although the different assays used to determine HLA-G expression, and the lack of a diagnostic methodology, induced some discrepancies between the results.

Melanoma is developed in melanocytes and its incidence is 11 in 100,000 (132). This cancer is not very aggressive, with a survival rate of 5 years in 81% of men and 87% of women (133). The expression of HLA-G in melanoma has been studied and demonstrated to be increased compared to melanocytic nevi (90), correlating HLA-G expression with cell transformation. HLA-G expression was also demonstrated to be increased on inflammatory infiltrating cells within the melanomas compared to nevus (134). sHLA-G was also increased in patients' sera seemingly being boosted by the IFN-α treatment applied (135). HLA-G expression was further shown to be associated to the malignant transformation and to bad prognosis, in case of metastasis or relapse, in different studies (128, 134, 136). Other groups demonstrated *in vitro* the immune-tolerogenic properties of HLA-G, protecting melanoma cell lines from the NK cells cytotoxicity (137), which were confirmed *in vivo* on xenogeneic melanoma models (56). Also, it was demonstrated that the tumor cells were able to modify their HLA-G isoform expression profile in order to modify their susceptibility against NK cells (44).

Gliomas represent 70% of the cerebral tumors, and their capability to modulate the immune response has been documented (138). The prognostic is usually bad since only 9.8% of the patients attain 5 years of survival after diagnosis. Wang et al. have reported that almost 70% of gliomas were HLA-G^+^ independently of their nature: oligodendroglioma, astrocytoma or oligoastrocytoma (139). Those results were confirmed by other groups (140). Wiendl et al. have widely studied the expression of HLA-G in cell lines derived from glioblastomas. They have demonstrated that 4 of 12 tumor cell lines constitutively expressed HLA-G mRNA. Following IFN-γ treatment, the number of gliomas expressing HLA-G mRNA dramatically increased since 10 out of 12 tumor cell lines were then HLA-G positive. Similar observations were stated for the HLA-G cell-surface expression (141). Other groups confirmed such results on gliomas using the demethylating agent 5-Aza-2′-Deoxycytidine (140). Strikingly, it was demonstrated that only 10% of glioblastoma cells expressing HLA-G were sufficient to inhibit the PBMCs alloresponse against the whole tumor (141). It has been widely demonstrated that an external stimulation is necessary to induce HLA-G expression at a transcriptional or translational level. In the context of glioblastomas, HLA-G expression was demonstrated to be influenced or regulated by environmental factors, particularly hypoxia or cytokines. Usually this environment is difficult to maintain *ex vivo*, where primary cells or cell lines loose rapidly their HLA-G expression, implying that the real expression levels of HLA-G in tumor cells are frequently underestimated (44).

Breast cancer represents 25.1% of female diagnosed cancers, and the 2nd most frequent of all cancers, with higher incidence in developed countries (142). There are three subtypes of breast cancer depending on the presence of 3 receptors for estrogen (ER), progesterone (PgR) and human epidermal growth factor (ERBB2/HER2). These different subtypes are mostly treated with chemotherapy or hormonal therapy. Ogiya et al. demonstrated that immune escape strategy of primary tumors is different from metastatic tumors in breast cancer. Primary tumors strategy involves higher infiltration of T cells expressing PD-L1 (143). Other studies indicate that secondary tumor focus present less immunoregulatory cells and weak expression of chemoattractants like CCL19/CCR7, CXCL9/ CXCR3, and IL15/IL15R (144). Nonetheless, the genomic and immune profile of a patient with triple-negative breast cancer that progressed during neoadjuvant chemotherapy plus PD-L1 blockade, showed a low level of expression of programmed cell death protein 1 (PD1) and a high level of expression of HLA-G at the time of diagnosis. This expression was associated with an immune evasive phenotype, increased cell motility and invasion, suggesting that HLA-G could be involved in tumor escape (145). Indeed, He et al. have studied the HLA-G expression in breast cancer and have determined that 66% of breast cancer cases are HLA-G positive, with a low HLA-G expression (<25% of tumor cells) in 64% of cases (146). This HLA-G expression on tumor cells is accompanied by the presence of sHLA-G in the sera and is associated with bad prognosis. It was also shown that there was an increase of circulating CD4^+^ CD25^+^ FoxP3^+^ T_reg_ cells in HLA-G^+^ patients compared with HLA-G^−^ patients (147). Other groups have shown that >60% were HLA-G^+^, from which >23% of cases co-expressed HLA-E (148, 149). Besides the loss of classical MHC class I expression, HLA-G and HLA-E expressions remained protecting tumor cells against NK cells cytotoxic response. Ishibashi et al. have reported higher expression rates with 94.1% of HLA-G^+^ tumor cells. They have demonstrated that a peptide derived from HLA-G1 (26–40 amino acid residues) was presented in the MHC class II context, inducing a CD4 response with consequent anti-HLA-G CTL detection. This was the first time that an anti-HLA-G cell response was ever reported (150). However, these results were never confirmed by other groups. Another study demonstrated that HLA-G expression was correlated to the double positive ER^+^/PgR^+^ tumors in 80% of cases (147). Previous studies had reported that HLA-G expression can be regulated by progesterone in mesenchymal cells, cytotrophoblast and choriocarcinoma cell line JEG3 (151, 152). Yet, the regulation via estrogens has never been reported.

Cervix cancer is the 2nd most frequent malign gynecologic cancer in the world representing 12% of female cancers (153). Pathogenesis is characterized by a progression of a cervical intraepithelial neoplasia (CIN) to cervix cancer (CC). Miranda et al. demonstrated that HLA-G is detected in 80.2% of CIN cases and in 64% of CC cases. Since the HLA-G expression level is higher in CC (48%) than in CIN (27%), it was suggested that HLA-G expression was correlated to the tumor development (154). HLA-G expression has also been correlated with IL-17 expression that could, on one hand, inhibit tumor progression by increasing the immune response, and on the other hand increase angiogenesis (155, 156). These results were confirmed by other groups who investigated HLA-G expression during the different stages of CIN. They concluded that HLA-G expression increased from 54% at CIN-I to 100% at CIN-IV, pointing out HLA-G as a good marker of the disease progression (157). Other groups also confirmed these results but percentages of HLA-G expression determined were weaker (158, 159). Guimaraes et al. demonstrated that HLA-G expression was highly correlated to human papilloma virus (HPV) in CC and inversely correlated to the MHC class I expression (158), confirmed by other group (159).

Serous epithelial ovarian cancer is the most common subtype of ovarian cancer (50–70% of ovarian cancer cases), followed by endometrioid carcinoma (10–25% of ovarian cancer cases). Diagnosis is frequently late given a mild symptomatology during first stages. This cancer is a serious carcinoma characterized by an aggressive development and bad prognosis. Endometrial carcinoma is the 3rd most frequent female cancer. This cancer is usually diagnosed at early stages and presents a favorable prognostic. HLA-G expression was demonstrated to be frequent in ovarian cancer (55%) with progression during disease development (160). HLA-G expression was evidenced at the transcriptional (qPCR) and translational levels (WB and IHC) with an increase from early stages (grade I/II) to late stages (grade III/IV) and a drop of survival rate of 5 years. Other groups have shown similar results, with higher HLA-G expression in serous carcinoma (161). HLA-G expression in endometrial cancer was studied by Barrier et al., who showed an expression of HLA-G mRNA in 55% of the cases of endometrioid cancers, mainly localized in the glandular epithelium with no expression was observed in the stromal tissue (162, 163), and the percentage of HLA-G^+^ lesions was also correlated with an advanced stage of the cancer.

Lung cancer is the most frequent malign cancer in the world, with an average of 800 000 deaths per year. There are two categories of lung cancer, (i) the small cell lung cancer (SCLC) which represents 10–15% of the cases, and (ii) the non-small cell lung cancer (NSCLC) that represents 85–90% of lung cancer cases. Despite some improvements in treatment, NSCLC remains a disease with bad prognosis. Indeed, the survival rate of 5 years is <15%. The most significant criteria to define the gravity and advanced stage of this cancer is the TNM state. Clinical observations and markers are still variable, so new markers are required to better define the stage of the disease. HLA-G expression has been proposed as one of such novel markers. Until now, all studies have been carried out in NSCLC for determining HLA-G expression. Yie et al. have demonstrated that 75% of the tumoral lesions they tested expressed HLA-G (164). HLA-G expression was considered as important (>50% of cells expressing HLA-G) in 80% of patients and was associated to the disease stage but independently of the histologic type lesion. HLA-G expression has also been correlated to a decrease in the survival rate. Other groups have confirmed these results not only by IHC, but also through sHLA-G dosage in patients' sera by ELISA (127). Western blot (WB) analysis demonstrated that the main HLA-G isoforms expressed were HLA-G1 and -G5. However, it seemed that sHLA-G origin was not from the tumor cell, but from peripheral blood monocytes (135, 165). Other authors demonstrated that sHLA-G was more frequently observed in adenocarcinoma (73%) than in epidermoid carcinomas (7%) or in adenosquamous carcinoma (10%). High HLA-G expression was determined in monocytes by in flow cytometry (166). In this context, Schütt et al proposed that membrane-bound HLA-G as well as sHLA-G were excellent progression markers (167) to be included as diagnosis markers.

## Discussion

The expression pattern of HLA-G on tumors is difficult to determine. The detection of HLA-G expressing cells, the nature of HLA-G isoforms and their impact on the immune system remain uncertain and challenging. Indeed, specific monoclonal antibodies are insufficient to define the isoforms concerned in the different type of tumors and involved in their developments. Furthermore, HLA-G expression tends to disappear after surgical excision of tumor lesion requiring to develop new culture approaches to maintain HLA-G expression *ex vivo* (44).

HLA-G mRNA expression can be determined by RT-PCR as previously reported (59, 168). However, mRNA expression is not directly correlated or associated with HLA-G protein expression (109), limiting the estimation of HLA-G actual expression in tumor tissue, particularly concerning the presence and percentage of the non-canonical isoforms, for which the antibodies (169) are scarce or inexistent (111). Indeed, regarding the detection of membrane-bound or secreted HLA-G isoforms (respectively, HLA-G1 to -G4 and HLA-G5 to -G7), few antibodies against HLA-G have been generated (Table 1). To overcome this limitation, a workshop to establish and standardize anti-HLA-G *in vitro* detection assays was initiated by the group of ED Carosella et al. (170, 171) and a wet workshop was organized for quantification and identification of soluble HLA-G (172). This allowed to determine HLA-G expression by immuno-histochemistry (IHC) western blot (WB), flow cytometry or ELISA assays in a more coordinated manner among laboratories. IHC and WB are essentially based on the utilization of the anti-HLA-G specific 4H84 and 5A6G7 monoclonal antibodies, whereas flow-cytometry and ELISA assays rely on 87G, MEM-G/9 and G233 monoclonal antibodies (mAbs). 4H84 mAb binds to the α1 domain (present in HLA-G1 to HLA-G7 isoforms) and the 5A6G7 mAb was raised against the intron 4 only present in secreted HLA-G isoforms (HLA-G5 to HLA-G7). Another antibody generated against denatured HLA-G is MEM-G/1, which can specifically detect denatured forms of HLA-G1 and -G2. Noteworthy, because MEM-G/1 targets an extracellular domain of native HLA-G which might be partially intrinsically disordered, this antibody not only can detect native forms of HLA-G2, but also competes with the LILRB2 binding of HLA-G2. These results provide novel insight into the functional characterization of HLA-G isoforms, pointing out its potential as ICP inhibitor (173). 87G, MEM-G/9 and G233 mAbs bind to conformational HLA-G α1 domain associated to the β2M (HLA-G1 and HLA-G5). However, immunoprecipitation assays on trophoblast surface demonstrated that G233 could detect a residual band of 39 kDa, either β2m-associated as well as a β2M-free heavy chain (174). Thus, determination of the HLA-G isoforms expressed is dependent on the combination of these different techniques. It must be pointed out that none of the anti-HLA-G antibodies generated were raised against the α2 or α3 domains. HLA-G sequence is strongly homolog to classical HLA molecules, particularly for the α2 and the α3 domains, and less for the α1 domain (175). This explains the bias for the α1 specificity of anti-HLA-G antibodies. Furthermore, the limited mAb development is also related to the fact that murine B cells express the PIR-B receptor, homolog to ILT2 or ILT4 human receptors, which inhibits murine B cell maturation and Ab secretion upon binding to HLA-G protein (66). Due to these limitations, experiments related to HLA-G expression and functions on tumor cells are not trivial. We have to point out that HLA-G expression studies are mainly performed on transfected or transduced tumor cell lines since HLA-G expression is rapidly loss after *ex vivo* culture or primary tumors. Conformational anti-HLA-G mAbs are limited to the HLA-G1/β2M or HLA-G5/β2M associated isoforms and to date, no mAb specific for the other HLA-G isoforms is available. Furthermore, the single blocking mAb to date against HLA-G is the 87G that only inhibits the function of HLA-G1/β2M or HLA-G5/β2m through ILT2 receptor. HLA-G2 and HLA-G6 isoforms are of interest since they are demonstrated to be immunosuppressive. Indeed, HLA-G2 and HLA-G6 isoforms encompass the α3 domain of HLA-G that mediates the interaction with the ILT4 inhibitory receptor expressed by APCs. Beyond this expression by immune cells, ILT4 was described on breast, lung and kidney tumor cells (113, 176, 177). Such site of expression, quite unexpected for ILT4, is of great interest with respect to how it affects the phenotypic and functional characteristics of tumor cells that express it (178). Neo-expression of ILT4 in breast cancer and in non-small cell lung cancer (NSCLC) is associated with metastasis in lymphatic nodes and poor prognostic (179). ILT4 expression is associated with an increase of cell proliferation and motility *in vitro* of tumor cells and promotes metastasis *in vivo* (180). Indeed, even if ILT4 is an inhibitory receptor, expressed on cancer cells, ILT4 inhibits mechanisms that repress proliferation, growth, and spread of cancer cells. Upon binding to its ligand, the ILT4 receptor inhibits the pathways that represses proliferation, growth and dissemination of tumor cells (181, 182). Since HLA-G is the main ligand of ILT4, HLA-G binding to ILT4 expressing cells, either by soluble HLA-G6 or membrane-bound HLA-G2 isoforms, could promote tumor growth. This heterogeneous expression of different ICPs within tumors, showed in the context of RCC (113), emphasizes the redundant or cumulative mechanisms developed by tumor cells to promote their immune escape and their expansion. Yet, mAbs capable of binding and/or blocking the ILT4 interaction with the conformational HLA-G2 and HLA-G6 isoforms are strongly lacking. Due to these drawbacks, tumor cell lines are essentially transduced with either HLA-G1 or HLA-G5 isoforms. Several tumors downregulate their MHC class I molecules expression at their surface by inhibiting the β2M expression to escape from the immune system (183–185). HLA-G cell surface expression on such tumors, even HLA-G1, could be unaffected by the loss of β2M association through the formation of HLA-G multimers as determined during pregnancy (186). Resulting β2M-free HLA-G isoforms could still be immunosuppressive and inhibit the immune response, particularly the NK immune response that should lyse MHC class I negative tumor cells. However, mAbs raised specifically against β2M-free HLA-G isoforms are lacking. In consequence, determination of the panel of HLA-G isoforms expressed by tumor cells is severely limited and the implications of β2M-free HLA-G isoforms in the tumor immune escape mechanisms are misestimated. Furthermore, Tronik-Le Roux et al. recently reported the expression of new HLA-G isoforms, devoid of α1 domain, but encompassing α2-α3 or α3 domains. As a fact, these new isoforms cannot be detected by the existing anti-HLA-G antibodies (111). Although HLA-G neo-expression has been proven in numerous tumors, it remains underestimated in most of the cancer lesions.

**Table 1:** Summary of the current available monoclonal antibodies raised against HLA-G isoforms.

**Designation**	**Specificity**	**Immunogen**	**References**
MEM-G/1	Denaturated heavy chain (α1 domain?)	Denaturated HLA-G1 heavy chain	(1–3)
MEM-G/2	Denaturated heavy chain (α1 domain?)	Denaturated HLA-G1 heavy chain	(4)
MEM-G/4	Denaturated heavy chain of HLA-G1, HLA-G2 and HLA-G5	Denaturated HLA-G1 heavy chain	(5)
MEM-G/9	Conformational HLA-G1/HLA-G5 isoforms associated with β2m	HLA-G recombinant protein refolded in presence of β2m and peptide	(5, 6)
G233	Conformational HLA-G1/HLA-G5 isoforms associated with β2m	Murine cells transfected with HLA-G1/β2m associated isoform	(7–9)
4H84	Denaturated heavy chain (α1 domain) of HLA-G1 to HLA-G7 isoforms	Peptide encompassing the amino acids 61-83 of HLA-G α1 domain	(4, 5, 10, 11)
5A6G7	Soluble isoforms HLA-G5 and HLA-G6	Peptide derived from intron 4 (SKEGDGGIMSVRESRSLSEDL) coupled with ovalbumin	(3, 12, 13)
2A12	Soluble isoforms HLA-G5 and HLA-G6	Peptide derived from intron 4 (SKEGDGGIMSVRESRSLSEDL) coupled with ovalbumin	(14, 15)
87G	Conformational HLA-G1/HLA-G5 isoforms associated with β2m and reported as blocking antibody	Murine cells transfected with HLA-G1/β2m associated isoform	(10, 16, 17)
HGY	Denaturated heavy chain (α1 domain?)	HLA-G purified proteins from placenta of pregnant women	(18, 19)

Here we emphasize the requirement of new tool development to analyze the HLA-G expression by tumor cells, especially the generation of new anti-HLA-G monoclonal antibodies to determine the expression pattern of HLA-G isoforms expressed by tumor cells.

It was suggested that HLA-G2/G6 may comprise an adequate substitute in women carrying the null allele (G^*^0105N) (187, 188). Also, it was demonstrated that melanoma cells can rapidly switch from cell-surface HLA-G1 to intra-cellular HLA-G2 expression, restoring tumor sensitivity to NK lysis (189). One can hypothesize that a switch between HLA-G isoforms expressed occurred following the development of the tumor. At the initial development stages, tumor cells would inhibit APC maturation and functions through ILT4 receptors by expressing HLA-G2 and HLA-G6 isoforms. Then, following angiogenesis and the tumor vascularization, effector cells that infiltrate the tumor would be inhibited by HLA-G1 and HLA-G5 isoforms through ILT2 receptors expressed on effector cells. This implies that depending on the stage of the tumor, different immunotherapies against HLA-G should be applied.

Since HLA-G/ILT2 and HLA-G/ILT4 are ICPs, inhibiting the interaction between immunosuppressive HLA-G isoforms and its receptors should restore the immune response as demonstrated for anti PD-1 and anti-PD-L1 monoclonal antibodies. Therefore, developing blocking antibodies against HLA-G/ILT4 and/or HLA-G/ILT2 interaction would restore the immune response. Wiendl et al. demonstrated that only 10% of tumor cells expressing HLA-G were enough to protect the whole tumor against the immune response (141). Thus, even if HLA-G expression is weak or diffuse within the tumor, the administration of anti-HLA-G blocking antibodies should dampen the immune-protective effects of HLA-G. LeMaoult et al. recently demonstrated that ccRCC tumors strongly expressed HLA-G and that the cytotoxic effector TILs were ILT2^+^ and PD-1^−^ (114). In this context, inhibiting the HLA-G/ILT2 interaction should restore the TILs cytotoxic function against HLA-G positive ccRCC tumors. As listed previously, HLA-G is an excellent TAA since HLA-G expression in healthy tissues is highly restrained, but strongly neo-expressed on tumors. As HLA-G expression level is correlated with an advanced stage of the disease, implying a decrease of the number of cytotoxic effector cells and their function, blocking antibodies would be insufficient in advanced stages. In this scenario, the cell therapies would be more adequate. Indeed, monoclonal anti-HLA-G antibodies could be used to develop anti-HLA-G CAR-T cells. These anti-HLA-G CAR-T cells would target directly and specifically the HLA-G expressing cells to eliminate the tumor.

Despite evidences that HLA-G expression is spread among hematopoietic and solid tumors, HLA-G expression is still largely underestimated. Insufficiency of biologic tools, in particular a wider specificity variety of anti-HLA-G monoclonal antibodies, make it difficult to determine and characterize HLA-G isoforms expressed, *de facto* limiting anti-HLA-G immunotherapies development.

## Author Contributions

MLo, JC, FA, MLe, and PL-D provided guidance and expertise in their respective areas of study. MLo, JC, and RD wrote the manuscript. All authors provided input, edited, and approved the final version of the manuscript.

## Conflict of Interest

The authors declare that the research was conducted in the absence of any commercial or financial relationships that could be construed as a potential conflict of interest.
